# MSSA-Net: Multi-Modal Structural and Semantic-Adaptive Network for Low-Light Image Enhancement

**DOI:** 10.3390/s26072059

**Published:** 2026-03-25

**Authors:** Tianxiang Chen, Xiaoyi Wang, Tongshun Zhang, Qiuzhan Zhou

**Affiliations:** 1 Samueli School of Engineering, University of California, Los Angeles, CA 90095, USA; 2 College of Computer Science and Technology, Jilin University, Changchun 130012, China; xiaoyiw23@mails.jlu.edu.cn (X.W.); tszhang23@mails.jlu.edu.cn (T.Z.); 3 College of Communication Engineering, Jilin University, Changchun 130012, China; zhouqz@jlu.edu.cn

**Keywords:** image sensor restoration, low-light enhancement, vision transformer

## Abstract

Low-light image enhancement (LLIE) remains challenging due to severe degradation of high-frequency structures and semantic ambiguity under extreme darkness. Although existing methods achieve satisfactory brightness recovery, they often suffer from structural inconsistency and semantic drift, as diverse scenes are typically processed with uniform enhancement strategies or static text prompts. To address these issues, we propose a Multi-Modal Structural and Semantic-Adaptive Network (MSSA-Net) under a structure-anchored paradigm. First, we design a Multi-Scale Self-Refinement Block (MSRB) to enhance degraded visible representations through multi-scale feature extraction and progressive refinement. Meanwhile, a pseudo-infrared structural prior derived from the input image is introduced to provide noise-insensitive geometric cues. These cues are extracted via a Structure-Guided Cross-Attention (SGCA) module to produce structure-dominant features. The refined visible features and structural features are then adaptively integrated through an adaptive residual fusion (ARF) module to achieve balanced restoration. Furthermore, we develop a Large Multi-modal Model (LMM)-Driven Scene-Adaptive Attention mechanism that generates instance-aware scene tags from a coarse preview and injects semantic embeddings into visual features. Extensive experiments demonstrate that MSSA-Net improves structural fidelity, brightness recovery, and semantic naturalness across multiple benchmarks.

## 1. Introduction

In the rapidly evolving landscape of computer vision and multimedia signal processing, the acquisition of high-fidelity visual data serves as the cornerstone for robust intelligent systems. Low-light image enhancement (LLIE) has transcended its traditional role as a mere aesthetic post-processing step to become a critical pre-requisite for safety-critical applications, including autonomous driving in tunnels [1], nocturnal video surveillance [2], and disaster search and rescue operations. However, in real-world uncontrolled environments, images captured by standard dynamic range sensors inevitably suffer from severe quality degradation. These degradations include low visibility, reduced contrast, and complex noise. The noise mainly arises from photon shot noise and read noise under low photon counts. Such corruption not only diminishes the subjective visual experience for human observers but also significantly reduces the accuracy of downstream machine vision algorithms, which heavily rely on clean gradient information for feature extraction.

The historical trajectory of LLIE research can be broadly categorized into histogram-based adjustments and Retinex-based physical models. Early approaches, such as Histogram Equalization (HE) and its adaptive variants [3], sought to statistically expand the dynamic range of pixel intensities. Concurrently, Retinex theory-based methods [4,5] decomposed images into reflectance and illumination components, attempting to recover the intrinsic scene properties under the idealized Lambertian assumption. Despite their theoretical elegance, these traditional paradigms often rely on hand-crafted priors that are too rigid for complex real-world scenarios. Consequently, they frequently introduce over-enhancement artifacts, noise amplification in dark regions, and color deviations when accurate atmospheric scattering models are unavailable.

In the deep learning era, the paradigm has shifted towards data-driven restoration. Convolutional Neural Networks (CNNs) [6,7] and, more recently, Transformers [8], have achieved remarkable quantitative strides by learning non-linear mappings from large-scale synthetic datasets. Unsupervised methods like Zero-DCE [9] have further relaxed the need for paired training data by formulating enhancement as a curve estimation task. While these spatial-domain methods effectively improve global brightness and contrast, a key limitation of current methods is that they predominantly rely on the visible light spectrum (RGB) alone and treat enhancement as a “black-box” signal-mapping process, ignoring the intrinsic structural and semantic entanglements characteristic of low-light scenes.

**Figure 1:**
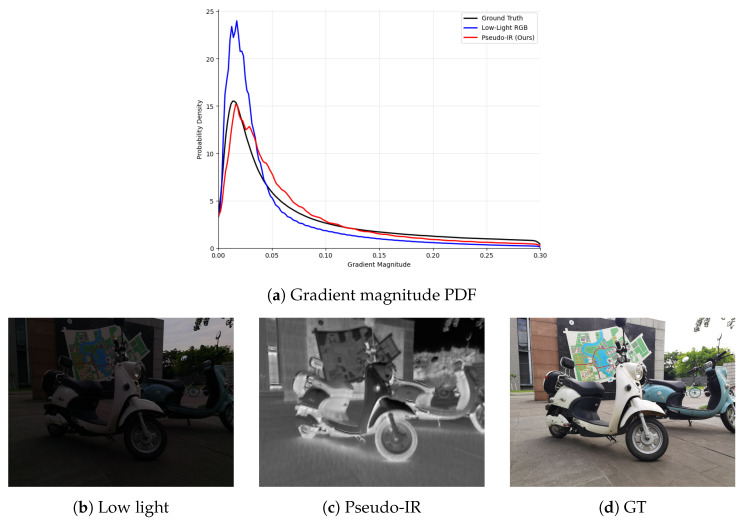
Statisticaland structural comparison between the pseudo-IR prior and the ground truth. (**a**) Gradient magnitude PDF; (**b**–**d**) visual results. The pseudo-IR prior aligns closer to the GT while suppressing noise.

We identify two fundamental bottlenecks inherent in RGB-only enhancement frameworks, where noise and informative features coexist within a single representation space, severely limiting robustness under extreme low-light conditions. First, the structural fidelity paradox. From a signal processing perspective, recovering high-frequency structures (edges and textures) from a low-signal-to-noise ratio (SNR) input is inherently ill-posed. As illustrated in Figure 1a, the gradient magnitude distribution of the raw low-light input exhibits a pronounced heavy tail and abnormal density in the mid-frequency range, deviating significantly from the Ground Truth. This excess density does not correspond to meaningful object boundaries but instead reflects stochastic noise contamination. As a result, valid structural contours become submerged within a chaotic gradient field. Consequently, deep networks trained solely on corrupted RGB inputs face a fundamental ambiguity: high-frequency responses may originate from either genuine edges or sensor noise. This ambiguity leads to a structural hallucination trap—where noise is over-sharpened into artificial details—or, conversely, to over-smoothed reconstructions that suppress fine structures. To resolve this entanglement, we reformulate the degraded RGB signal into a noise-insensitive structural domain. While hardware infrared (IR) imaging naturally offers structural stability, it is constrained by cost and cross-modal misalignment. Instead, we generate a pseudo-infrared structural prior by re-projecting the input into a geometry-oriented representation (as shown in Figure 1c). This transformation does not introduce external data; rather, it suppresses non-structural stochastic noise while preserving deterministic boundary cues. The resulting pseudo-infrared map serves as a clean structural anchor for the restoration stream, enabling the model to distinguish authentic geometric contours from noise. By imposing this structural constraint, our framework prevents noise amplification and preserves fine textures with improved fidelity.

**Figure 2:**
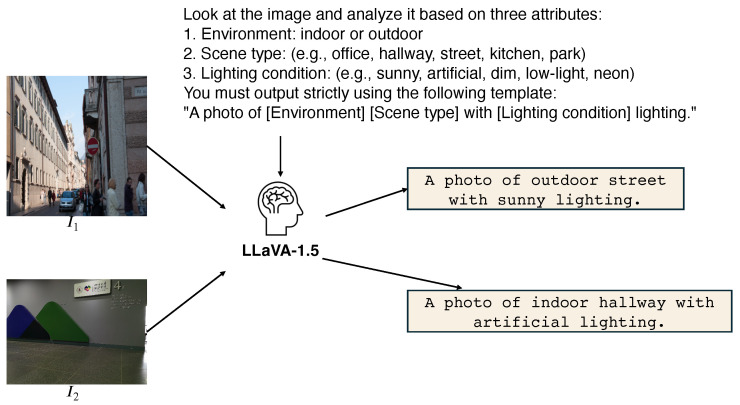
Scene-level semantic description generated by LLaVA.

Second, the semantic ambiguity dilemma. The mapping from a low-light image to a normal-light image is not one-to-one but one-to-many, heavily dependent on the scene’s semantic context. A pixel with low intensity could represent a naturally black object in daylight or a white object in total darkness. Without high-level semantic constraints, standard “blind” enhancement models apply a uniform processing strategy across diverse scenes, resulting in semantic drift. For instance, aggressively whitening a warm, tungsten-lit indoor scene destroys its intrinsic atmosphere, while under-enhancing a moonlit road compromises safety. Although recent Vision–Language Model (VLM) approaches [10] attempt to introduce text guidance, they typically rely on static, generic prompts (e.g., “enhance this image”), failing to capture the nuanced photometric characteristics of specific scenes (e.g., “sunny” vs. “artificial lighting”). As illustrated in Figure 2, we argue that effective enhancement requires adaptation to scene semantics, dynamically adapting its restoration strategy based on the specific semantic content of the scene.

To dismantle the structural and semantic limitations of RGB-only enhancement, we propose a Multi-Modal Structural and Semantic-Adaptive Network (MSSA-Net) under a structure-anchored, semantically-navigated paradigm. First, we introduce a Multi-Scale Self-Refinement Block (MSRB) to enhance intrinsic visible representations under extreme low-light conditions. MSRB performs multi-scale feature extraction with global–local context aggregation, enabling self refinement while suppressing noise contamination. Building upon the refined visible features, we generate a structural pseudo-infrared prior and integrate it via a Structure-Guided Cross-Attention (SGCA) module. SGCA injects noise-insensitive geometric cues from the pseudo-infrared domain into the visible stream, explicitly guiding boundary reconstruction and alleviating structural ambiguity caused by low-SNR inputs. To reconcile the complementary yet heterogeneous structure-dominant and texture-dominant features, we further design an adaptive residual fusion (ARF) module. ARF employs a spatial gating mechanism to adaptively aggregate cross-modal information. Finally, to address semantic ambiguity at a higher semantic level, we develop an LMM-Driven Scene-Adaptive Attention (LSAA) module. LSAA generates instance-aware scene description from a coarse preview, encodes them into token-level semantic embeddings, and injects them into visual features via cross-attention. This dynamic semantic conditioning calibrates illumination and color adjustment according to scene context, improving scene-awareness and semantic consistency during enhancement. By jointly integrating multi-scale self refinement, structural anchoring, adaptive fusion, and dynamic semantic conditioning, MSSA-Net achieves coordinated optimization of brightness recovery, structural fidelity, and perceptual coherence.

The main contributions of this work are summarized as follows:

- We propose a novel multi-modal framework, termed MSSA-Net, which integrates a pseudo-infrared structural prior with LMM-based semantic conditioning under a structure-anchored paradigm. By jointly modeling geometric and semantic cues, the framework alleviates the representation ambiguity inherent in RGB-only low-light enhancement.
- We introduce a pseudo-infrared anchoring strategy implemented via the SGCA module, which reformulates structural representation into a noise-insensitive domain. This mechanism improves geometric consistency and stabilizes boundary reconstruction under low-SNR conditions.
- We design an LMM-Driven Scene-Adaptive Attention (LSAA) module that generates instance-aware scene descriptions and injects token-level semantic embeddings into visual features via cross-attention. This dynamic semantic conditioning enhances scene-awareness and improves cross-scene generalization during low-light enhancement.

## 2. Related Work

### 2.1. Low-Light Image Enhancement

LLIE has progressed from physics-based priors to deep learning models. Early CNN-based methods like RetinexNet [6] and Zero-DCE [9] utilized decomposition and zero-reference curve estimation, respectively, while EnlightenGAN [11] introduced unpaired adversarial training. Recently, Transformers have shown superior long-range dependency modeling. Restormer [12] employs channel-wise attention for restoration, and Retinexformer [8] integrates physical priors to suppress noise. Despite these advances, single-representation methods often encounter performance bottlenecks when structural information is obscured by heavy noise [13].

### 2.2. Multi-Modal Image Restoration

To compensate for information loss, multi-modal fusion utilizes auxiliary signals like infrared or depth. DeepFuse [14] and U2Fusion [15] propose unified frameworks to fuse features for geometric preservation. PIAFusion [16] further aligns infrared and visible features based on illumination conditions. While effective for structure, these methods often rely on simple concatenation or addition, leading to feature misalignment or texture pollution in the final restored image.

### 2.3. Vision–Language and Semantic Guidance

Integrating high-level semantics ensures perceptual naturalness. Semantic-aware methods [17] leverage segmentation maps to guide region-specific enhancement. With the advent of Vision–Language Models (VLMs), CLIP-Lit [10] and InstructIR [18] utilize text embeddings to steer restoration. However, these approaches typically depend on static, generic prompts, failing to adapt to dynamic scene contexts.

## 3. Method

### 3.1. Multi-Modal Structural and Semantic-Adaptive Framework

**Figure 3:**
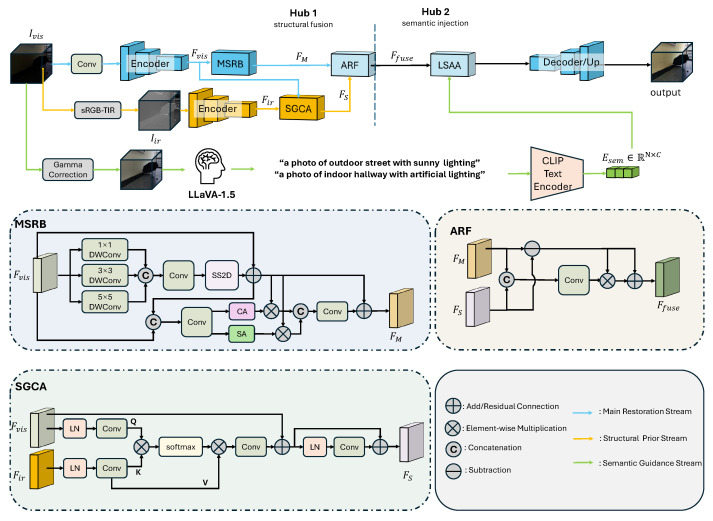
Our framework first utilizes a SGCA module to inject infrared priors, while a MSRB captures intrinsic details. These complementary features are integrated via ARF, and finally, the restoration is calibrated by the LSAA to ensure semantic consistency.

Existing low-light enhancement methods often treat all scenes uniformly, neglecting scene-dependent lighting characteristics (e.g., backlit corridors versus uneven artificial illumination). To address this limitation, we propose the Multi-Modal Structural and Semantic-Adaptive Network (MSSA-Net), a multi-modal framework that disentangles low-light degradation into structural recovery and content restoration, while being explicitly conditioned on scene semantics. As illustrated in Figure 3, our framework operates on two streams: the main restoration stream and the structural prior stream. Given a low-light RGB input $I_{vis}$, we first employ a pre-trained sRGB-to-TIR translation [19] to generate a pseudo-infrared image $I_{ir}$. The model is trained on paired RGB–thermal data and is kept frozen during both training and inference. While derived from the same sensor input, the pseudo-infrared modality functions as a cross-representation prior. Since the generator is fixed, the pseudo-infrared prior provides a stable structural reference without introducing additional optimization variables. It highlights gradient-invariant structures that are typically suppressed in the RGB space, thereby guiding the network to bypass the limitations of a single-stream learning. Since thermal representations are less sensitive to illumination noise, the generated modality provides robust geometric and edge priors. The structural prior stream extracts structural features from $I_{ir}$, while the main restoration stream processes $I_{vis}$ to recover latent color distributions and fine-grained textures suppressed under low illumination. Rather than directly concatenating features—which often causes cross-modal misalignment—we adopt a hierarchical interaction strategy in which pseudo-infrared structural priors progressively guide visible feature restoration across multiple scales. This dual-guidance design ensures geometrically consistent reconstruction while preserving detailed textures. Beyond structural modeling, we further recognize that low-light enhancement requires semantic awareness to maintain perceptual realism. Therefore, we introduce a Scene-Adaptive Attention mechanism driven by a Large Multi-modal Model (LMM). A coarsely enhanced preview is first analyzed to generate a scene description (e.g., “A photo of outdoor street with sunny lighting,” “A photo of indoor hallway with artificial lighting”), which is then encoded using the pre-trained CLIP model [20]. The resulting semantic embeddings dynamically modulate visual features, enabling scene-adaptive enhancement strategies. By jointly leveraging structure guidance and semantic context conditioning, MSSA-Net achieves a balanced restoration that preserves structural fidelity while maintaining perceptual naturalness.

#### 3.1.1. Multi-Scale Self-Refinement Block (MSRB)

Firstly, in order to recover the intrinsic texture details of the visible image, we propose the Multi-Scale Self-Refinement Block (MSRB), which consists of a multi-scale extraction branch followed by a global–local context modeling unit.

##### Multi-Scale Feature Extraction

To accommodate features at different scales, the input passes through three parallel branches with depthwise convolutions of varying kernel sizes (1×1, 3×3, and 5×5). This design allows the network to simultaneously preserve fine textures and suppress large-scale noise. The multi-scale features are concatenated and fused to obtain the intermediate feature $F_{multi}$:

(1) $$F_{multi}={\mathrm{Conv}}_{1\times 1}\left(\mathrm{Concat}[{\mathrm{DWConv}}_{1\times 1}\left(F\right),{\mathrm{DWConv}}_{3\times 3}\left(F\right),{\mathrm{DWConv}}_{5\times 5}\left(F\right)]\right)$$

##### Global–Local Context Modeling

Convolutional networks often struggle with long-range dependencies. To address this, we process $F_{multi}$ through a 2D Selective Scan (SS2D) module [21], which employs a spatial-shifting mechanism to capture global interactions with linear complexity. Subsequently, to refine local features, we apply parallel Channel Attention (CA) and Spatial Attention (SA) mechanisms. These attention modules adaptively recalibrate the features along channel and spatial dimensions, highlighting informative regions while suppressing noise. The outputs are concatenated and fused to produce the final refined feature $F_{M}$:

(2) $$F_{M}=\mathrm{Conv}\left(\mathrm{Concat}[\mathrm{CA}\left(F^{\prime }\right),\mathrm{SA}\left(F^{\prime }\right)]\right)+F^{\prime }$$

where $F^{\prime }$ denotes the features after the SS2D operation. $F_{M}$ serves as the final texture-enhanced representation ready for the fusion stage.

#### 3.1.2. Structure-Guided Cross Attention (SGCA)

In extreme low-light environments, the visible image features $F_{vis}$ suffer from severe noise and blurred boundaries, whereas the pseudo-infrared features $F_{ir}$ preserve clear structural details. To effectively transfer these structural priors, we design the Structure-Guided Cross-Attention (SGCA) module.

Unlike standard self-attention mechanisms, we formulate the noisy visible features as the Queryto “search” for structural guidance, while the reliable infrared features serve as the Key and Value. Specifically, given the intermediate feature maps $F_{vis},F_{ir}\in R^{H\times W\times C}$, we first apply Layer Normalization (LN) and project them into query (*Q*), key (*K*), and value (*V*) embeddings using depthwise separable convolutions to capture local spatial context:

(3) $$Q=W_{d}^{Q}\left(\mathrm{LN}\left(F_{vis}\right)\right),\;K=W_{d}^{K}\left(\mathrm{LN}\left(F_{ir}\right)\right),\;V=W_{d}^{V}\left(\mathrm{LN}\left(F_{ir}\right)\right)$$

where $W_{d}^{\left(\cdot \right)}$ denotes the depthwise projection layers. The attention map is calculated to measure the structural correlation, and the retrieved structural cues are aggregated:

(4) $$\mathrm{Attention}\left(Q,K,V\right)=\mathrm{Softmax}\left(\frac{QK^{T}}{\sqrt{d_{k}}}\right)V$$

Here, $\sqrt{d_{k}}$ is a scaling factor to stabilize gradients. The structure-enhanced feature $F_{S}$ is obtained via a residual connection:

(5) $$F_{S}=\mathrm{Conv}\left(\mathrm{Attention}\left(Q,K,V\right)\right)+F_{vis}$$

This mechanism ensures that the restoration is guided by reliable structural boundaries, preventing the hallucination of artifacts commonly found in single-modal methods. Although the pseudo-infrared representation is generated and may contain estimation errors, the proposed cross-attention formulation alleviates their impact. Since the visible features act as queries, the network is encouraged to selectively attend to more reliable structural cues from the pseudo-infrared features rather than directly enforcing them. As a result, potential noise or inconsistencies in the pseudo-infrared prior are less likely to adversely affect the restoration process.

#### 3.1.3. Adaptive Residual Fusion (ARF)

The proposed network generates two complementary feature representations: the structure-dominant features $F_{S}$ from the SGCA module (rich in boundary information) and the texture-dominant features $F_{M}$ from the MSRB module (rich in visible details). Direct summation or concatenation of these features may lead to redundancy or feature misalignment. To achieve an effective synthesis, we design the adaptive residual fusion (ARF) module.

ARF employs a spatial gating mechanism to selectively aggregate information based on regional importance. We first concatenate the two features and pass them through a convolution layer followed by a Sigmoid activation function to generate a spatial weight map *M*:

(6) $$M=\sigma \left({\mathrm{Conv}}_{1\times 1}\left(\mathrm{Concat}\left[F_{S},F_{M}\right]\right)\right)$$

The gating map *M* encodes pixel-wise modulation weights that adaptively measure the structural–textural discrepancy at each spatial location. Instead of directly blending the two representations, ARF performs a residual modulation centered on $F_{M}$:

(7) $$F_{fuse}=M\times \left(F_{S}-F_{M}\right)+F_{M}$$

This formulation allows the network to selectively amplify or suppress the differential components between the two modalities, achieving adaptive compensation instead of direct aggregation. In this way, structural guidance refines texture representation in a spatially adaptive manner, leading to a more coherent unified feature.

Finally, a residual block is applied to further stabilize optimization and enhance feature consistency.

#### 3.1.4. LMM-Driven Scene-Adaptive Attention (LSAA)

**Figure 4:**
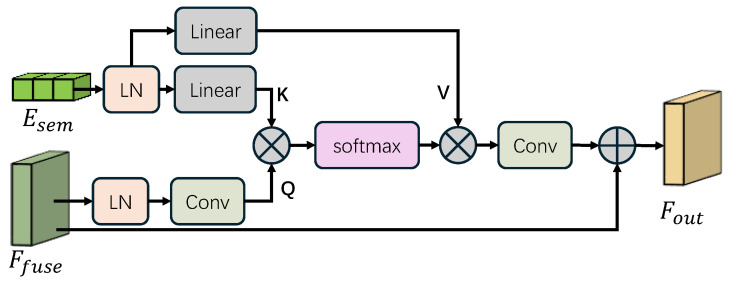
LSAA aggregates dynamic semantic cues and visual features via cross-attention.

Traditional text-guided methods rely on fixed prompts (e.g., “restore the image”), which fail to adapt to the semantic diversity of real-world scenes. To achieve instance-level adaptability, we propose the LMM-Driven Scene-Adaptive Attention (LSAA). As shown in Figure 4, this module dynamically calibrates the network’s features based on the high-level semantic content of the scene.

##### Scene-Adaptive Description Generation

Since the input image is extremely dark, directly feeding it to a Large Multi-modal Model (LMM) may yield inaccurate descriptions. We first apply a lightweight brightening operation (e.g., gamma correction) to obtain a coarse preview $I_{coarse}$. We then leverage a pre-trained LMM (e.g., LLaVA-1.5 [22]) as a semantic interpreter. Instead of generating long, ambiguous sentences, we instruct the LMM via prompt engineering to output a scene-adaptive description for the image. Our prompt is as follows:

Look at the image and analyze it based on three attributes:

1. Environment: indoor or outdoor;
2. Scene type: (e.g., office, hallway, street, kitchen, park);
3. Lighting condition: (e.g., sunny, artificial, dim, low-light, neon).

You must output strictly using the following template: “A photo of [Environment] [Scene type] with [Lighting condition] lighting.”

Examples of valid outputs:

- A photo of indoor office with artificial lighting.
- A photo of outdoor street with sunny lighting.

Rules:

- Only output the final constructed sentence.
- Do not add explanations, introductory text, or quotation marks.
- Do not output the isolated words.

This constrained template ensures deterministic and concise outputs, avoiding ambiguity in semantic descriptions.

##### Semantic Feature Cross-Attention

The constructed sentence is then tokenized and encoded by a frozen CLIP Text Encoder to produce a token-level semantic sequence embedding $E_{\mathrm{sem}}\in R^{N\times C}$, where *N* denotes the token length and *C* represents the channel dimension. This robust embedding encapsulates the high-level understanding of the scene (e.g., recognizing that “artificial” requires a colder color temperature than “sunny”). To explicitly inject this guidance into the visual representation, we employ a cross-attention mechanism.

Specifically, the fused visual features $F_{fuse}$ serve as the spatial queries to retrieve relevant semantic context, while the semantic embedding $E_{sem}$ acts as the keys and values. We first apply Layer Normalization (LN) to both modalities. The query (*Q*) is generated via a convolution layer to preserve spatial structures, whereas the key (*K*) and value (*V*) are obtained through independent linear projections:

(8) $$Q=\mathrm{Conv}\left(\mathrm{LN}\left(F_{fuse}\right)\right),\;K=\mathrm{Linear}\left(\mathrm{LN}\left(E_{sem}\right)\right),\;V=\mathrm{Linear}\left(\mathrm{LN}\left(E_{sem}\right)\right)$$

The cross-attention is then computed to dynamically aggregate semantic cues into every spatial location of the visual features. The output is projected via another convolution layer and added to the original visual features through a residual connection to produce the semantically calibrated features $F_{out}$:

(9) $$F_{out}=\mathrm{Conv}\left(\mathrm{Softmax}\left(\frac{QK^{T}}{\sqrt{d_{k}}}\right)V\right)+F_{fuse}$$

where $d_{k}$ is the scaling factor to stabilize gradients. By explicitly injecting scene semantics via cross-attention, LSAA guides the restoration network to apply context-specific enhancement strategies, effectively reducing color deviation and artifacts caused by blind enhancement.

### 3.2. Loss Function

To train MSSA-Net, we employ a composite loss function to ensure both pixel-level fidelity and semantic naturalness:

(10) $$L_{total}=\lambda_{1}L_{rec}+\lambda_{2}L_{struct}+\lambda_{3}L_{vgg}+\lambda_{4}L_{clip}$$

We set $\lambda_{1},\lambda_{2},\lambda_{3},\lambda_{4}=\left[1.0,0.1,0.2,0.01\right]$, where $L_{rec}$ is given by ${∥I_{\mathrm{out}}-I_{\mathrm{gt}}∥}_{2}$ and $L_{struct}$ is the SSIM [23] loss for structural integrity. $L_{vgg}$ denotes the VGG-based [24] perceptual loss. Additionally, $L_{clip}$ is the semantic consistency loss, calculated as the cosine similarity between the CLIP embeddings of the enhanced image and the LMM-generated tags, ensuring the final output visually aligns with the predicted scene description.

## 4. Experiments

### 4.1. Datasets and Experimental Setting

MSSA-Net is trained and evaluated on three LLIE datasets: LOL-v2-Real, LOL-v2-Synthesis, and LSRW-Huawei. LOL-v2-Real provides 689 training and 100 testing pairs with more diverse real-world scenarios. LOL-v2-Synthesis includes 900 training and 100 testing synthesized pairs. LSRW-Huawei comprises 2450 training and 30 testing pairs captured with different devices. Since these datasets contain only RGB images, we generate the corresponding pseudo-infrared images using [19] to train our structure-aware branch. The pre-trained translation model is directly applied without further fine-tuning. Implemented in Pytorch 2.6, MSSA-Net is trained end-to-end to optimize network parameters jointly. During training, images are augmented with random horizontal/vertical flips and rotations. We use Adam optimizer with $\beta_{1}=0.9,\beta_{2}=0.99$, and an initial learning rate of $3\times 10^{-4}$. Training is conducted for a total of $1.5\times 10^{5}$ iterations with a batch size of 8. Regarding the LMM implementation in the LSAA module, we employ the LLaVA-1.5-7B model [22]) as the core semantic engine. The LMM is used in inference-only mode and remains frozen without any gradient updates during training. To minimize the computational footprint and GPU memory requirements, we adopt 4-bit quantization via the BitsAndBytes framework, and constrain the max number of generated tokens to 10. The scene-adaptive tags are generated only once at the beginning of the enhancement process (for both training and inference stages) based on a downsampled coarse preview. The same prompt template is consistently used during both training and inference to ensure reproducibility and stable semantic conditioning. The generated semantic description is reused throughout the entire restoration process without repeated LMM inference, which significantly reduces computational overhead. On a single NVIDIA RTX 3090 GPU (Nvidia, Santa Clara, CA, USA), the average inference latency for the LMM to generate a concise tag set is approximately 0.2 s per image. Since these semantic tags remain constant throughout the subsequent multi-scale restoration iterations, the overall throughput of MSSA-Net remains competitive for practical applications.

### 4.2. Comparison with Current Methods

We compare our method with various SOTA methods for low-light enhancement, including LIME [5], SRIE [25], Kind [26], MIRNet [27], Kind++ [28], SNR-Aware [13], RetinexFormer [8], UHDFour [29], UHDFormer [30], DMFourLLIE [31], URKWV [32] and RetinexMamba [33].

#### 4.2.1. Quantitative Results

To objectively validate the efficacy of MSSA-Net, we employ three widely recognized full-reference metrics: Peak signal-to-noise ratio (PSNR), Structural Similarity Index (SSIM), and Learned Perceptual Image Patch Similarity (LPIPS) [34]. PSNR serves as a vital indicator of signal fidelity, where a higher value signifies more effective suppression of noise. Meanwhile, SSIM offers a more sophisticated evaluation by quantifying the preservation of luminance, contrast, and structural information, thereby aligning more closely with human visual perception. In addition, LPIPS measures perceptual similarity in a deep feature space, where a lower value indicates better alignment with human visual judgment in terms of texture and semantic consistency.

As summarized in Table 1, MSSA-Net achieves consistently competitive performance across multiple benchmarks. While the numerical improvements over recent methods are relatively modest in some cases, the proposed framework demonstrates better stability and robustness, particularly in semantic consistency, which is further corroborated by consistently lower LPIPS scores. On the LOL-v2-Real dataset, our model achieves a leading PSNR of 23.25 dB and an SSIM of 0.87, along with the lowest LPIPS score of 0.049, outperforming recent state-of-the-art methods such as Retinexformer [8] and DMFourLLIE [31]. This improvement in perceptual similarity highlights the effectiveness of our model in preserving realistic textures under complex real-world noise. For the LOL-v2-Synthesis dataset, MSSA-Net reaches a pinnacle SSIM of 0.94, achieving high structural similartiy, while also achieving a competitive LPIPS score of 0.027, second only to DMFourLLIE [31]. Furthermore, on the highly challenging LSRW-Huawei benchmark—where traditional methods like LIME [5] and SRIE [25] fail significantly with SSIM scores below 0.50—our method maintains a superior SSIM of 0.64 and achieves the best LPIPS score of 0.153. Although DMFourLLIE [31] shows strong performance, MSSA-Net still manages to yield a lower LPIPS and competitive SSIM, indicating its effectiveness in preserving high-frequency textual strokes, edge integrity, and perceptual realism.

#### 4.2.2. Visualization Comparison

**Figure 5:**
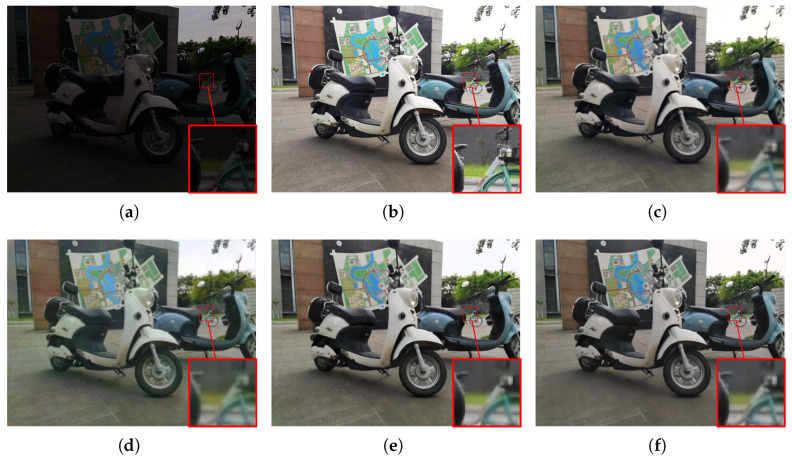
Visual comparison with state-of-the-art methods on the LSRW-Huawei dataset. (**a**) Input. (**b**) Reference. (**c**) UHDFormer. (**d**) UHDFour. (**e**) DMFourLLIE. (**f**) MSSA-Net.

**Figure 6:**
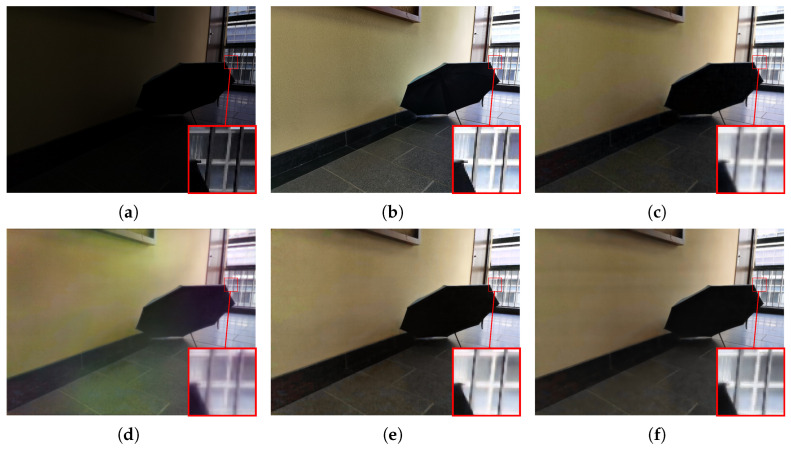
Visual comparison with state-of-the-art methods on the LSRW-Huawei dataset. (**a**) Input. (**b**) Reference. (**c**) UHDFormer. (**d**) UHDFour. (**e**) DMFourLLIE. (**f**) MSSA-Net.

**Figure 7:**
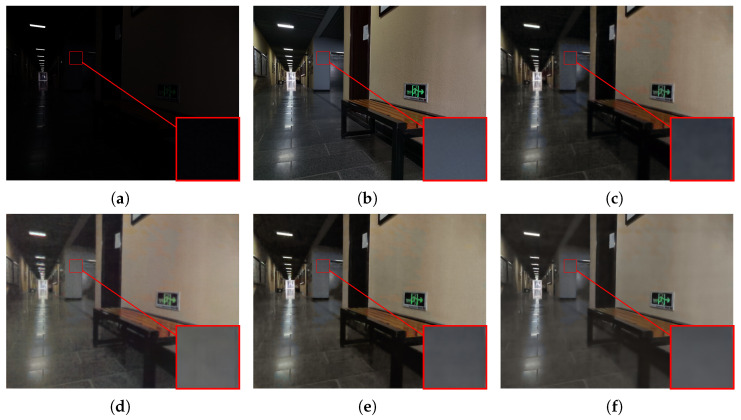
Visual comparison with state-of-the-art methods on the LSRW-Huawei dataset. (**a**) Input. (**b**) Reference. (**c**) UHDFormer. (**d**) UHDFour. (**e**) DMFourLLIE. (**f**) MSSA-Net.

**Figure 8:**
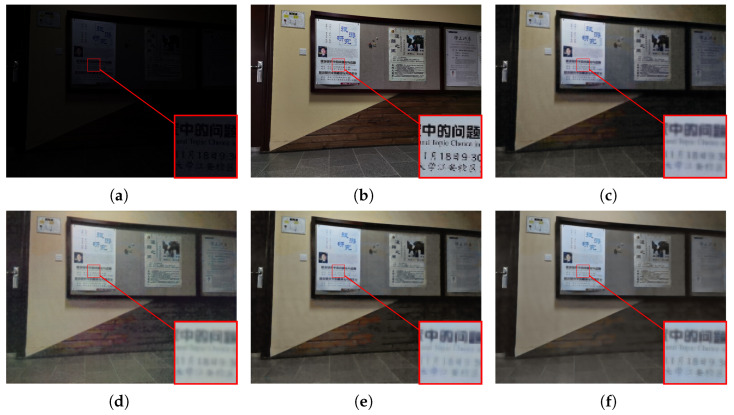
Visual comparison with state-of-the-art methods on the LSRW-Huawei dataset. (**a**) Input. (**b**) Reference. (**c**) UHDFormer. (**d**) UHDFour. (**e**) DMFourLLIE. (**f**) MSSA-Net.

To subjectively verify the enhancement performance, we conduct a detailed comparative analysis between the proposed MSSA-Net and several state-of-the-art methods, including UHDFormer [30], UHDFour [29], and DMFourLLIE [31]. As illustrated in Figure 5, where the input image is characterized by extreme darkness and non-uniform illumination, UHDFour [29] tends to produce excessive brightness lifting in shadow areas, which compromises the natural contrast and results in a washed-out appearance with flattened visual depth. Similar artifacts persist in Figure 6, where the complex edge and corner structures pose significant challenges; both UHDFour [29] and DMFourLLIE [31] consistently exhibit varying degrees of over-enhancement and ringing artifacts around the boundaries, whereas UHDFormer [30] fails to provide sufficient brightness, leaving several regions under-enhanced and obscured. In contrast, MSSA-Net achieves precise exposure calibration in Figure 6, ensuring that the luminance levels are optimized for human visual perception without introducing artificial glare or corner distortions. Our method shows improved results in Figure 7, where the large-area homogeneous wall reveals the weaknesses of competitors; while UHDFour [29] and DMFourLLIE [31] struggle with chromatic aberration and unnatural color mottling on the wall surface, MSSA-Net achieves more accurate illumination estimation and vibrant chromatic consistency that is closely aligned with GT. Furthermore, observing the results in Figure 8, which contains fine text details, most competing algorithms, including UHDFormer [30], UHDFour [29], and DMFourLLIE [31], suffer from noticeable blurring, character smearing, and structural distortions, rendering the text nearly illegible. In sharp contrast, MSSA-Net preserves sharper edges and fine-grained textual strokes, effectively mitigating distortions and delivering high-fidelity results that are consistently superior to other state-of-the-art methods across diverse and challenging low-light scenarios.

### 4.3. Ablation Study

To validate the effectiveness of each component and our architectural design choices, we conduct systematic ablation studies. The experiments are divided into component integration, module replacement, and loss function analysis.

#### 4.3.1. Component Integration

We evaluate the model in a progressive integration manner, gradually incorporating multi-scale refinement, structural guidance, and semantic attention to examine their individual and combined effects, as reported in Table 2. Starting with a single-branch baseline containing only the MSRB module, the network achieves a base PSNR of 21.55 dB. When we bypass the MSRB and only employ the structural guidance (SGCA) on the unrefined visible features, the performance drops to 21.32 dB. This clearly indicates that the ARF fusion module requires the high-quality, multi-scale features extracted by MSRB to function effectively. By integrating both MSRB and SGCA, the dual-branch architecture leverages structural prior, lifting the PSNR to 22.23 dB. Finally, the full model (ours), which incorporates the LSAA module to inject textual semantic priors, achieves the best performance (23.25 dB). This step-wise progression proves that our modules operate synergistically, jointly modeling visible details, structural prior, and semantic context.

#### 4.3.2. Component Replacement and Semantic Priors

In Table 3 and Table 4, we analyze the effect of architectural substitutions and luminance preprocessing on semantic-guided restoration.

Replacing the cross-modal guided attention and adaptive residual fusion with naive feature concatenation (SGCA → Concat) and element-wise summation (ARF → Sum) leads to sub-optimal performance (22.27 dB and 22.43 dB, respectively). This demonstrates that simple aggregation strategies are insufficient for effectively aligning and integrating cross-modal representations.

Regarding semantic guidance, substituting the dynamic scene-aware prompt with a fixed description such as “restore the image” (Text → Fixed) limits adaptability and reduces performance to 22.25 dB. Feeding a null token (Text → Null) further degrades the result to 21.34 dB, highlighting the importance of meaningful semantic conditioning for stable cross-attention interactions.

We further examine the role of gamma correction before the LMM. Directly feeding extremely dark inputs without gamma correction significantly degrades performance (22.85 dB). Applying moderate gamma correction substantially improves restoration quality, with γ=0.3 achieving the best result, while γ=0.1 and γ=0.5 exhibit slightly lower yet comparable performance. These results suggest that luminance adjustment facilitates more consistent semantic alignment, whereas insufficient or excessive enhancement may slightly affect restoration fidelity. Overall, gamma correction acts as a simple yet effective step to improve semantic robustness and downstream restoration performance.

#### 4.3.3. Loss Function and Hyper-Parameter Sensitivity

The results in Table 5 demonstrate the sensitivity of MSSA-Net to different loss weight configurations and confirm that our default setting ($\lambda_{rec}=1.0$, $\lambda_{struct}=0.1$, $\lambda_{vgg}=0.2$, $\lambda_{clip}=0.01$) achieves the optimal performance, reaching 23.25 dB in PSNR and 0.87 in SSIM.

Firstly, the experimental data reveals that the structural loss ($\lambda_{struct}$) is a pivotal component for maintaining geometric fidelity. When $\lambda_{struct}$ is reduced to 0.05, we observe a sharp performance drop to 22.63 dB, which is the lowest PSNR recorded in our ablation study. This underscores the critical role of structural constraints in recovering sharp edges and fine details from low-light degradations. Conversely, an overly rigid structural constraint (e.g., $\lambda_{struct}=0.2$) also leads to a decline (22.86 dB), suggesting that excessive regularization may limit the model’s flexibility in restoring pixel intensities.

Secondly, the sensitivity analysis of the reconstruction loss ($\lambda_{rec}$) and perceptual-related losses ($\lambda_{vgg},\lambda_{clip}$) further validates the robustness of our default strategy. As shown in Table 5, either decreasing $\lambda_{rec}$ to 0.5 or increasing it to 2.0 results in sub-optimal performance. This indicates that a balanced pixel-wise constraint is essential: insufficient weighting leads to poor convergence, while excessive focus on pixel-wise minimization may cause the model to overfit to local noise or artifacts. Similarly, deviations in $\lambda_{vgg}$ and $\lambda_{clip}$ from their default values result in lower quantitative metrics, as these terms must be meticulously balanced to harmonize high-level semantic coherence with low-level image fidelity. In summary, our weighting strategy provides the most effective trade-off between visual quality and quantitative accuracy.

### 4.4. High-Level Vision Task Evaluation

**Figure 9:**
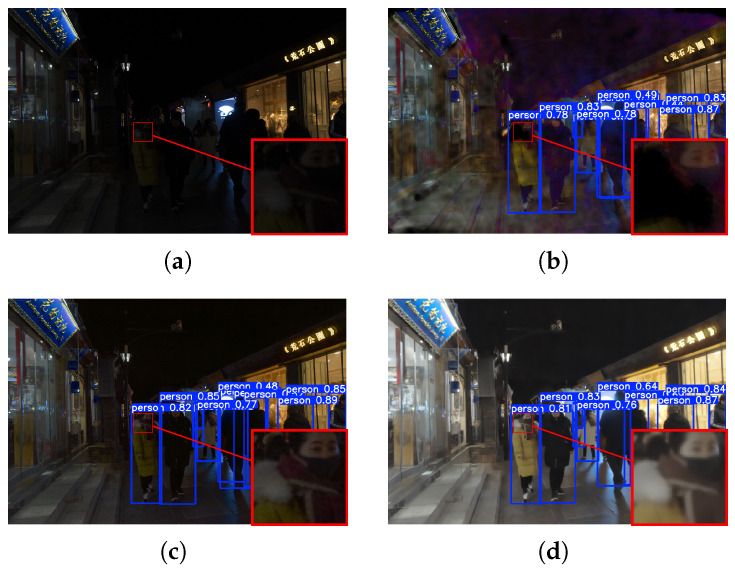
Comparison of dense face detection results under complex scene conditions. (**a**) Input. (**b**) DMF. (**c**) UHDFormer. (**d**) MSSA.

**Figure 10:**
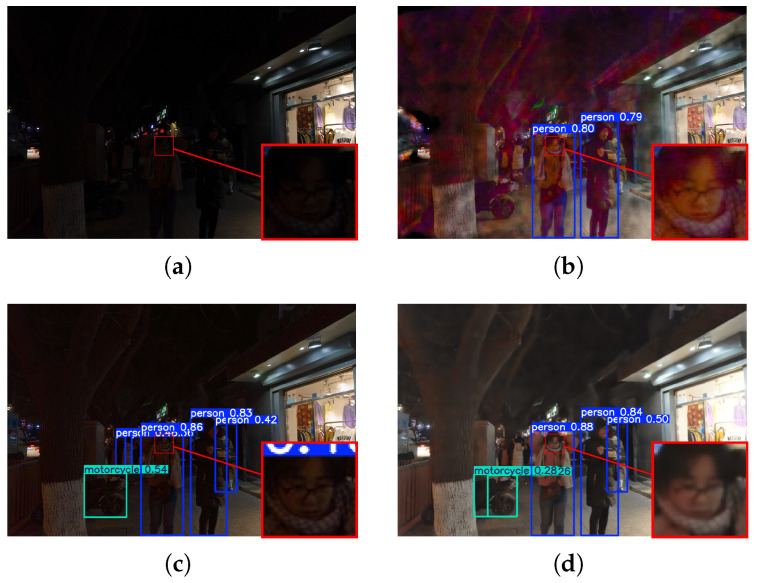
Comparison of dense face detection results under complex scene conditions. (**a**) Input. (**b**) DMF. (**c**) UHDFormer. (**d**) MSSA.

The practical value of low-light image enhancement lies in its ability to facilitate subsequent machine-oriented perception tasks. To validate the robustness of our framework in real-world scenarios, we conduct face detection experiments on the DarkFace dataset [35] using a pre-trained YOLOv5 detector. Visual comparisons in Figure 9 and Figure 10 reveal that existing methods often struggle with the zero-shot generalization required for extreme darkness. Specifically, results from UHDFormer remain under-exposed, failing to provide sufficient illumination for distant or small-scale targets, while DMFourLLIE introduces noticeable artifacts that interfere with feature extraction and lead to false negatives. In contrast, our method achieves a better balance between brightness restoration and noise suppression, providing cleaner textures and more reliable semantic information. Reflecting these visual improvements, our approach achieves a higher mean detection confidence of 0.5623, surpassing recent state-of-the-art models such as UHDFormer (0.5591) and DMFourLLIE (0.5484). These results demonstrate that our framework can serve as a robust and effective front-end for automated vision systems in complex nighttime environments.

## 5. Conclusions

We propose MSSA-Net, a novel multi-modal low-light enhancement framework that integrates a pseudo-infrared structural prior with LMM-based semantic conditioning under a structure-anchored paradigm. By jointly modeling geometric and semantic cues, the framework alleviates the representation ambiguity inherent in RGB-only enhancement. The network adopts a dual-branch design, where a Multi-Scale Self-Refinement Block enhances degraded visible representations and a Structure-Guided Cross-Attention branch introduces structurally stable boundary cues to improve geometric consistency under challenging lighting conditions. An adaptive residual fusion module performs region-aware feature integration, while the LMM-Driven Scene-Adaptive Attention module injects scene-level semantic constraints to promote perceptually coherent enhancement. Extensive experiments demonstrate that MSSA-Net achieves consistently competitive performance across multiple benchmarks in both quantitative and visual evaluations. Future work will explore richer semantic descriptors and extend the proposed structure-anchored framework to video enhancement and other challenging restoration tasks.

## Figures and Tables

**Table 1:** Results on LOL-v2 and LSRW-Huawei datasets of different models.

Methods	LOL-v2-Real			LOL-v2-Sync			LSRW-Huawei		
	PSNR	SSIM	LPIPS	PSNR	SSIM	LPIPS	PSNR	SSIM	LPIPS
LIME	15.24	0.42	0.220	16.88	0.76	0.104	17.00	0.38	0.207
SRIE	14.45	0.52	0.216	14.50	0.66	0.148	13.42	0.43	0.217
Kind	17.54	0.67	0.081	22.62	0.90	0.052	16.58	0.57	0.226
MIRNet	22.11	0.79	0.145	22.52	0.90	0.057	19.98	0.61	0.215
Kind++	19.08	0.82	0.088	21.17	0.88	0.068	15.43	0.57	0.237
SNR-Aware	21.48	0.85	0.074	24.13	0.93	0.032	20.67	0.59	0.192
UHDFour	21.79	0.85	0.115	23.60	0.91	0.034	19.39	0.60	0.247
RetinexMamba	22.45	0.84	0.055	25.88	0.93	0.039	20.88	0.63	0.169
Retinexformer	22.80	0.84	0.072	25.67	0.93	0.029	21.23	0.63	0.170
URKWV	23.11	**0.87**	0.054	**26.36**	**0.94**	0.042	20.92	**0.64**	0.192
DMFourLLIE	22.64	0.86	0.052	25.83	0.93	**0.025**	21.47	0.63	0.180
MSSA-Net	**23.25**	**0.87**	**0.049**	25.84	**0.94**	0.027	**21.42**	**0.64**	**0.153**

Bold indicates the best performance.

**Table 2:** Step-wise component integration ablation study.

MSRB	SGCA (IR)	LSAA (Text)	PSNR	SSIM
✓	x	x	21.55	0.84
✓	x	✓	22.12	0.85
x	✓	x	21.32	0.84
x	✓	✓	21.78	0.85
✓	✓	x	22.23	0.86
✓	✓	✓	**23.25**	**0.87**

✓ and x denote the presence and absence of the corresponding component respectively. Bold indicates the best performance.

**Table 3:** Ablation study of component replacement.

Setting	PSNR	SSIM
SGCA → Concat	22.27	0.86
ARF → Sum	22.43	0.86
Text → Fixed	22.25	0.85
Text → Null	21.34	0.85
Full Model	**23.25**	**0.87**

Bold indicates the best performance.

**Table 4:** Sensitivity analysis of gamma correction.

Gamma	PSNR	SSIM
w/o Gamma	22.85	0.86
γ=0.1	23.08	0.86
γ=0.3	**23.25**	**0.87**
γ=0.5	23.02	0.86

Bold indicates the best performance.

**Table 5:** Ablation study of loss components and hyper-parameter sensitivity.

$\lambda_{rec}$	$\lambda_{struct}$	$\lambda_{vgg}$	$\lambda_{clip}$	PSNR	SSIM
1.0	0.1	0.2	0.01	**23.25**	**0.87**
0.5	0.1	0.2	0.01	23.01	0.86
2.0	0.1	0.2	0.01	23.15	0.85
1.0	0.05	0.2	0.01	22.63	0.85
1.0	0.2	0.2	0.01	22.86	0.86
1.0	0.1	0.1	0.01	22.85	0.86
1.0	0.1	0.4	0.01	22.95	0.85
1.0	0.1	0.2	0.005	23.16	0.86
1.0	0.1	0.2	0.02	23.12	0.86

Bold indicates the best performance.

## Data Availability

The original contributions presented in this study are included in the article. Further inquiries can be directed to the corresponding author.
